# The MicroRNAs as Prognostic Biomarkers for Survival in Esophageal Cancer: A Meta-Analysis

**DOI:** 10.1155/2014/523979

**Published:** 2014-07-06

**Authors:** Wenbo Fu, Lijuan Pang, Yunzhao Chen, Lan Yang, Janbo Zhu, Yutao Wei

**Affiliations:** ^1^Medical College of Shihezi University, Shihezi, Xinjiang 832000, China; ^2^Department of Thoracic and Cardiovascular Surgery, Hospital of Xinjiang Production and Construction Corps, Urumchi, Xinjiang 830002, China; ^3^Department of Pathology, Medical College of Shihezi University, Shihezi, Xinjiang 832000, China; ^4^Department of Thoracic and Cardiovascular Surgery, First Hospital Affiliated to Medical College of Shihezi University, Shihezi, Xinjiang 832000, China

## Abstract

*Objectives.* We performed this meta-analysis to summarize all the results from available studies, aiming delineating the prognostic role of miRNA in esophageal cancer.* Design and Methods*. We searched the electronic databases PubMed, EMBASE, and ISI Web of Science without time restrictions for the correlative literature to aggregate the survival results. Relevant data were extracted from studies investigating the relationship between miRNAs expression and survival in esophageal cancer patients. Pooled hazard ratios of miR-21and miR-375 for OS in ESCC were calculated.* Results.* A total of 25 studies involving 2,258 subjects analyzed the relationship between miRNA and prognosis of EC. In all, thirty-nine miRNAs associated with prognosis were reported in these studies. The pooled HR of higher miR-21 expression compared with lower miR-21 expression in ESCC was 1.84 (95% CI: 1.41–2.40, *P* < 0.001), which could significantly predict poorer OS in ESCC. Besides, higher miR-375 was also a significant predictor for OS in ESCC, with a pooled HR of 0.55 (95% CI: 0.42–0.72, *P* < 0.001).* Conclusions.* Our results support that miR-21 and miR-375 have a prognostic role in ESCC and may be useful therapeutic targets for the treatment of ESCC and meticulous follow-up for early detection of recurrence.

## 1. Introduction

Esophageal cancer (EC) represents an interesting but a very lethal group of malignancies associated with varied risk factors and geographical distribution [1]. EC is the sixth most common cancer and the sixth leading cause of cancer death in human worldwide, affecting men more than women [2]. According to a new study, there were estimated 482,300 new EC cases and 406,800 EC-caused deaths in 2008 worldwide [3]. The deadly disease is often diagnosed at later stages, and the survival rate for affected patients is very low, ranging from 10% in Europe [4] to 20–30% in Asia [5], so that the prognosis of affected patients is unsatisfactory, despite the development of therapeutic options such as surgery, chemotherapy, and radiotherapy, highlighting the need to identify novel biomarkers for early detection and prognostic classification [6].

Recently, microRNAs (miRNAs) have attracted attention for their involvement in cell development, differentiation, proliferation, and apoptosis by targeting mRNAs for cleavage or translational repression at the posttranscriptional level [7]. The miRNAs are a class of small, approximately 23 nucleotide noncoding RNAs that are highly conserved and endogenously expressed across mammals and other species [8]. These little molecules are transcribed in the nucleus and are subsequently cleaved by the class II RNase III enzyme Drosha to produce pre-miRNAs [9]; these pre-miRNAs are then exported to the cytoplasm where the RNase III enzyme Dicer further processes them into mature miRNAs [7]. They act as part of a multiprotein complex to silence or repress the expression of target genes [10] and have emerged as a key component of posttranscriptional regulation [11]. Calin et al. [12] demonstrated that more than 50% of miRNA genes are located in fragile sites and cancer-associated genomic regions, suggesting that miRNAs may play a vital role in the pathogenesis of human cancers and are able to dramatically change the biological function of organisms. Growing evidence has indicated that aberrant expression of miRNAs has been linked to development and progression of cancer and has been shown to have prognostic significance in several tumor types, including colon [13], lung [14], breast [15], and ovarian cancer [16]. Recent studies showed that miRNAs are associated with prognosis in EC as well, suggesting that they could be developed as prognostic classifiers to guide therapeutic decisions [17, 18]. We performed the meta-analysis of the data available from articles published in this field with the main aim of evaluating the role of specific microRNA as a prognostic biomarker in EC.

## 2. Material and Methods 

This meta-analysis followed the guidelines of the Meta-analysis of Observational Studies in Epidemiology group (MOOSE) issued by Stroup et al. [19] and Preferred Reporting Items for Systematic Reviews and Meta-analysis (PRISMA) criteria by Moher et al. [20].

### 2.1. Publication Search

We carefully searched the PubMed, EMBASE, and ISI Web of Science databases using the search terms “miRNA,” “microRNA,” “esophageal cancer,” “esophageal neoplasm,” “esophageal carcinoma,” “ESCC,” “prognos∗,” and “surviv∗” updated until January 15, 2014, and limited to English language papers. Furthermore, reference lists of retrieved articles and review articles were reviewed manually to identify missing relevant publications.

### 2.2. Eligibility Criteria

Two reviewers (Pang and Chen) independently assessed eligibility of the retrieved articles. The studies were included in the meta-analysis if they reported survival data in esophageal cancer patients, provided sufficient data for determining an estimate of HR and a 95% CI, and enrolled more than 20 patients, and study patients did not overlap with patients in other included articles. We did not assign each study a quality score, because no such score has received general agreement for use in a prognostic meta-analysis, especially of observational studies [21].

### 2.3. Data Extraction

Two reviewers (Zhu and Wei) independently extracted the data of the included studies. The following data was extracted: name of first author, year of publication, country, number of participants, study design, tumor stage, HR with 95% CI, and so forth. In the study reported by Zhao and colleagues [17], no HR or 95% CI were available; thus the HR and 95% CI were estimated from the *P* value of the log rank test and the number of observed events in each group, or the Kaplan-Meier curve [22].

### 2.4. Statistical Analyses

For miRNAs evaluated appropriately in a single study, the summary HR (including 95% CI) represents the value reported in that study. For miRNAs that were evaluated appropriately in multiple studies, fixed effects summary HR and 95% CI were calculated by using the generic inverse variance method and the random effects model according to the D-L method [23]. Interstudy heterogeneity was assessed by the *I* ^2^ statistic [24]. By convention, an observed HR > 1 indicated worse outcome for the study group relative to the reference group and would be considered statistically significant if the 95% confidence interval did not overlap 1, with *P* < 0.05 [25]. Articles that presented insufficient data to derive the HR and CI for OS and PFS/RFS were included in the systematic review (qualitative analysis) but excluded from the meta-analysis (quantitative analysis) [26]. Publication bias was weighted by Begg's funnel plot with the Egger's bias indicator test with *P* < 0.05 being considered statistically significant [27]. All of the analyses were performed using STATA v11.0 (Stata Corp., College Station, TX) [28].

## 3. Result

Two-hundred and eighty-seven studies for miRNAs associated with prognosis were identified from a primary literature search in PubMed, EMBASE, and ISI Web of Science databases. After manually screening the titles, abstracts, and key data, two-hundred and sixty-six records were excluded because they were either duplicate, review articles, or irrelevant to the current study. Twenty-seven articles were further reviewed in detail. Two articles were further excluded because data were unavailable for further analysis [29, 30]. A total of 25 studies involving 2,258 subjects analyzed the relationship between miRNA and prognosis of EC (data was not shown). In all, thirty-nine miRNAs associated with prognosis were reported in these studies and miR-21, miR-145, and miR-375 were reported by more than three articles. There are four studies investigating the relationship between miR-145 and prognosis of EC. Pooled HR and 95% CI for miR-21/375 to predict OS in ESCC were calculated. Data were not synthesized for miR-145 because of different EC histology and types of survival analysis. Clinicopathological characteristics of eligible studies are summarized in Table 1. For miR-21, 5 of 7 studies were conducted in Asian population, 1 derived from multipopulation, and 1 study was performed in western population, which may lead to a discrepancy of ethnicity. One study included 50 patients, 3 studies examined between 51 and 100 individuals, and 3 studies examined between 100 and 200 individuals. Cancerous tissues were usually examined to determine miRNAs expression level, while plasma or serum samples were also tested in two studies [31, 32]. For miR-375, 4 of 6 studies were conducted in Asian population, 1 derived from multipopulation, and 1 study was performed in western population. One study included 50 patients, 2 studies examined between 51 and 100 individuals, and 3 studies examined between 100 and 200 individuals. Four studies investigate the relationship between miR-145 and prognosis of EC. All of the studies were retrospective in design. Notably, the cutoff values of miRNAs were different in each study, most with median or mean. More detailed information was summarized in Table 1.

**Figure 1:**
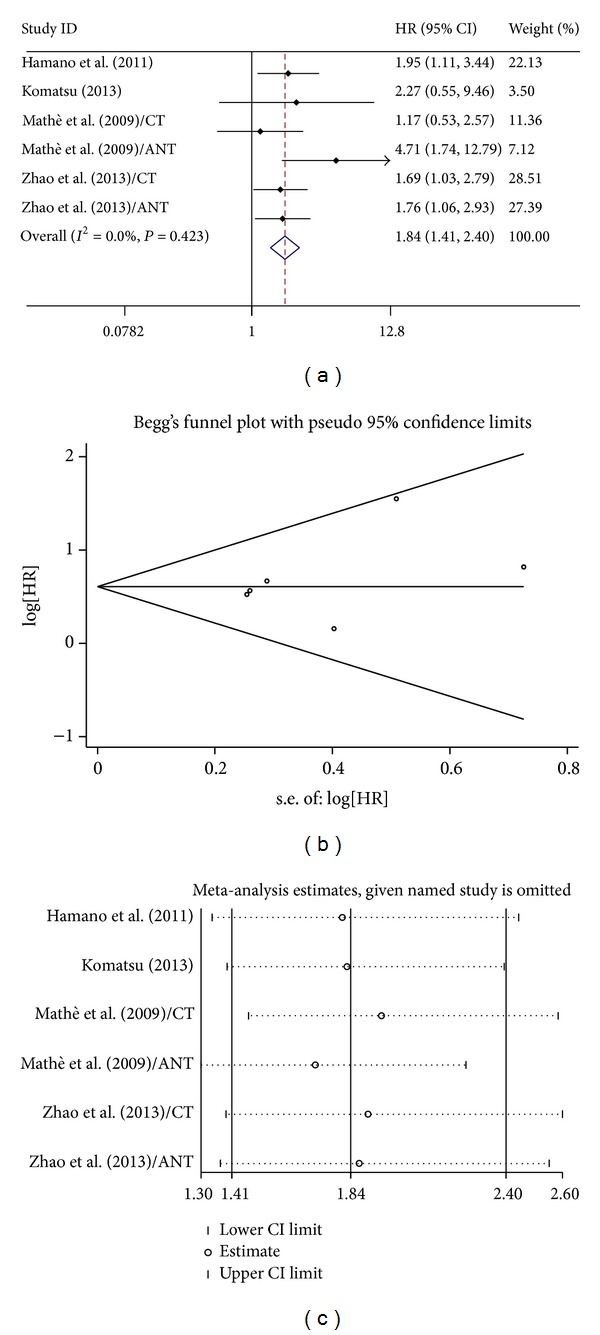
(a) Forest plots of studies evaluating HR of survivals comparing high and low miR-21 expression. (b) Funnel plots of publication bias for meta-analysis of miR-21. (c) Sensitivity analysis for meta-analysis of miR-21.

**Figure 2:**
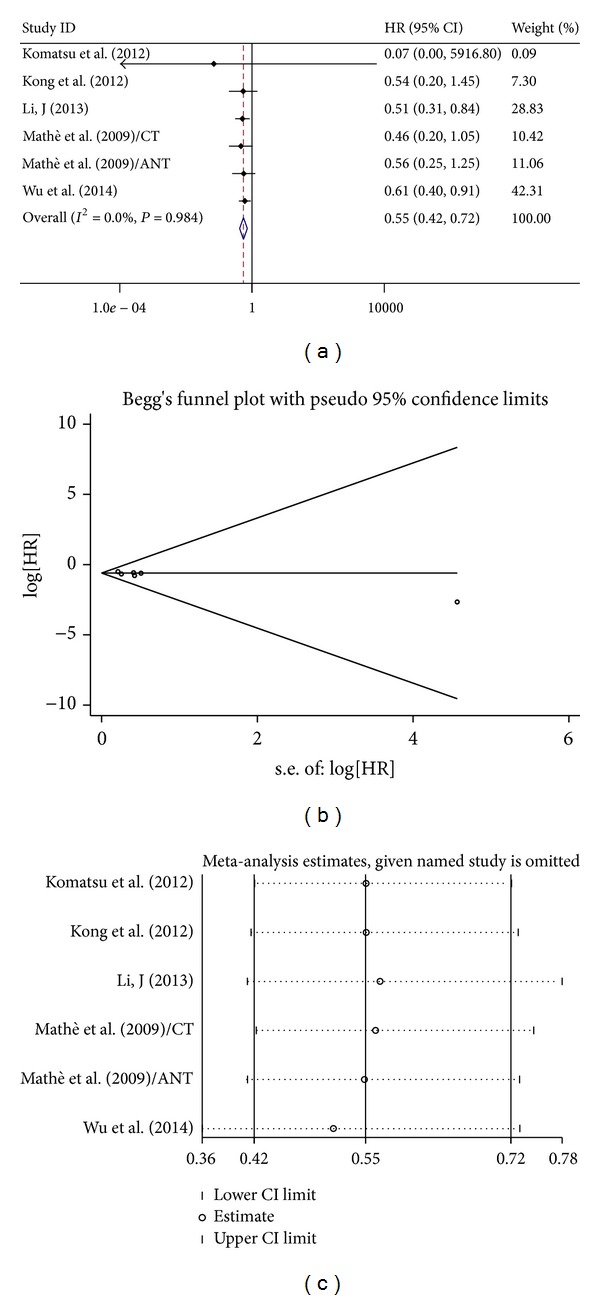
(a) Forest plots of studies evaluating hazard ratios (HRs) of high miR375 expression as compared to low expression. (b) Funnel plots of publication bias for meta-analysis of miR-375. (c) Sensitivity analysis for meta-analysis of miR-375.

For studies evaluating OS in ESCC, no significant heterogeneity have been found (for miR-21: *I* ^2^ = 0.00%, *P* = 0.26; miR-375: *I* ^2^ = 0.00%, *P* = 0.13), so that the fixed effects were applied to calculate the pooled HR (pooled HR = 1.84, 95% CI 1.41–2.40 *P* < 0.001 for miR-21, Figure 1; and 0.55, 95% CI: 0.42–0.72, *P* < 0.001 for miR-375, Figure 2). Publication bias of the included studies was evaluated by Begg's plots and Egger's test. No significant publication biases were found in the results of Begg's plots of miR-21 and miR-375. Egger test reached similar conclusions. Pooled HR and 95% CI were not calculated for miR-145 because of different EC histology and types of survival analysis.

## 4. Discussion

In response to the need for independently prognostic molecular markers for ESCC that are readily assayable on routinely acquired clinical specimens, we conducted this analysis of the published esophageal cancer literature to identify a group of miRNAs for which the data support validation as prognostic biomarkers of esophageal cancer outcomes. In this first extensive systematic review about miRNAs in esophageal cancer, 25 studies involving 2,258 subjects analyzed the relationship between miRNA and prognosis of esophageal cancer (data was not shown). A total of 39 miRNAs involved in the prognosis of esophageal cancer were reported in these studies. Most miRNAs were identified by one single study and only 3 miRNAs (miR-21,375,145) were reported by more than 3 studies and only miR-21/375 was suitable for pooled analysis. Wang et al. [33] systematically analyzed miR-21 expression in 16 pairs of ESCC and the corresponding matched nonmalignant adjacent tissues. The author showed that miR-21 downregulation inhibits cell growth and invasion and induces cells to apoptosis by targeting FASL, TIMP3, and RECK genes. Interestingly, suppression or knockdown of miR-21 could induce apoptosis and repress cell proliferation and invasion [34, 35]. One conclusion of the Hamano's [34] study was that miRNAs (including miR-21) that regulate stem cell function are involved in resistance to chemotherapy in esophageal cancer [34]. Ma et al. [35] investigated the role of microRNA-21 (miR-21) and its regulation on phosphatase and tensin homolog deleted from chromosome-10 (PTEN) in esophageal cancer and demonstrated that miR-21 was overexpressed in vitro and ESCC patients, and promoted cell proliferation by targeting PTEN at posttranscriptional level. Furthermore, miR-21 is known as an oncogenic miRNA that is increased in esophageal cancer, and overexpression of miR-21 plays important roles in increasing cell proliferation, migration, invasion, and survival [33, 36]. The meta-analysis showed that elevated miR-21 (pooled HR = 1.84) expression indeed predict poor OS in patients with ESCC. Although the number of associated studies dealing with miR-21 was less than 8, the available data have shown that miR-21 was associated with the prognosis of ESCC, malignant tumor of the digestion system. However, the conclusions should be tempered because the pooled risks of miR-21 for survival, although statistically significant, were not strong, with global HRs of 1.84. The promoter of miR-375 was frequently hypermethylated in EC and miR-375 is a negative regulator of 3-phosphoinositide-dependent protein kinase-1 (PDK1) in EC [37]. Besides, the downregulation of miR-375 was frequently detected in primary ESCC, which was significantly correlated with advanced stage, distant metastasis. MiR-375 could interact with the 3′-untranslated region of IGF1R and downregulate its expression. Functional assays demonstrated that miR-375 could inhibit clonogenicity, cell motility, cell proliferation, tumor formation, and metastasis in mice [38]. This finding is consistent with Mathe's [39] finding that reduced levels of miR-375 are associated with worse prognosis. Pooled HR of upregulated miR-375 for OS in ESCC patients were 0.55, indicating that low level of miR-375 has a negative impact on OS. The predicted targets of miR-145 were oncogenes myc myelocytomatosis viral-related oncogene (MYCN), FOS, and yamaguchi sarcoma oncogene (YES); cell-cycle promoters such as cyclins D2 and L1; and mitogen-activated protein kinase (MAPK) transduction proteins such as MAP3K3 and MAP4K4 [40]. Kano et al. [41] revealed that miR-145 inhibits cell proliferation and invasion when overexpressed in esophageal carcinoma cell lines in vitro, possibly through downregulation of the FSCN-1 gene product. The transfection of human esophageal carcinoma cells with miR-145 expression plasmids resulted in a greater inhibition of cell mobility; however, the protein level of the previously reported target of miR-145, FSCN1 did not show any significant downregulation [42]. Intriguingly, patients with high levels of miR-145 in the posttreatment biopsy specimens had significantly shorter median disease-free survival (DFS) than did those with low levels [43]. The result is consistent with Feber's [44] finding that high expression of miR-145 is associated with worse survival. Meta-analysis was not performed for miR-145 due to the fact that different EC histology and survival analysis were analysed. The role of miR-145 in EC prognosis remains unclear, although the included studies suggested that miR-145 could be a potential prognostic biomarker for esophageal cancer. Therefore we strongly suggest conducting more prognostic studies for abnormal expression of miR-145 in esophageal carcinomas.

There were several limitations inherent to our analysis. First, although miR-21 has been widely investigated, unfortunately, only seven studies were eligible for pooled analysis. Because of a lack of studies, the limited sample size, and missing data, we could not evaluate subgroups for the sample type, race, and many other factors, especially AD. Furthermore, miR-21 has been found upregulated in patients with non-small cell lung cancer, breast cancer [45], pancreatic cancer [46], and hematological malignancies [47]. Therefore, miR-21 may be a general biomarker for cancer rather than specific for esophageal cancer, although the level of expression may still have prognostic benefits. Second, since the number of studies for miR-375 was less than 7, it might weaken the reliability of our conclusion. A well-designed clinical study with large cases of each miRNAs should be performed in the future to validate the relationship between miR-21/375/145 expression level and prognosis of EC patients. Third, a clear definition should be made about the cutoff value of miRNAs level for outcomes in ESCC. To date, most investigators use median or mean value in their studies as the cutoff value and the accurate value were different. Fourth, although there was no significant evidence of publication bias in this analysis, cautions should be taken because only studies published in English were selected, which could definitely cause language bias. And the tendency for journals to publish positive results could also make certain bias [28]. Furthermore, tissues are more widely used for miRNA study. However, circulating markers are more acceptable than tissue markers because they can be assayed before treatment and be monitored throughout the life [17]. MiRNAs are considerably resistant to RNase digestion. Also, samples treated under harsh conditions, such as boiling, low/high pH, extended storage, and freeze-thaw cycles, yielded no significant differences compared to nontreated serum; thereby they are ideal biomarkers for clinical usage [48]. Three studies [31, 32, 49] included in this analysis examined the miRNAs expression levels in in serum/plasma. Thus, the simultaneous detection of miRNAs might serve as a tool to assess the “real-time” status of patients. It is hoped that detection of miRNAs during patient follow-ups would offer the opportunity for early intervention. More studies should be conducted in future to evaluate the prognostic value of miRNA level in serum/plasma. Besides, a group of miRNAs might be better than a single miRNA. Zhao et al. [17] suggest that expression patterns of miR-21, miR-181b, and miR-146b, alone or in combination with inflammatory risk score can be used as prognostic classifiers for patients with ESCC [17]. Komatsu et al. [32] revealed that the presence of high miR-21 and low miR-375 concentrations in plasma was an independent prognostic factor. The study of a set of miRNAs associated with prognosis for esophageal cancer is certainly warranted.

## 5. Conclusion

Despite the limitations described above, our results support that miR-21 and miR-375 have a prognostic role in ESCC and therefore may be useful therapeutic targets for the treatment of ESCC and meticulous follow-up for early detection of recurrence. Validation of the results of these miRNAs by independent and prospective study is certainly warranted, and it is hoped that this analysis highlights the most appropriate candidates for their clinical significance and role in the prognosis of esophageal cancer.

## Figures and Tables

**Table 1:** Clinicopathological characteristics of eligible studies.

miRNA	Study	Year	Population	Sample	Histology	*N*	Stage	PT (Y/N/U)	Sample collection	Control group	Gender (M/F)	Age	Method	Kits	EC	Cutoff	HR	Result
miR-375	Komatsu	2012	Japan	Plasma	ESCC	50	I–IV	0/50/0	Prior to surgery	normal	44/6	≥65, 25; <65, 25	qRT-PCR	TaqMan	U6	Normal	R	OS
	Kong	2012	China	Frozen	ESCC	60	I–IV	0/60/0	During resection	normal	43/17	≤66, 23; >66, 37	qRT-PCR	SYBR	SNORD48	Normal	SC	OS/DFS
	Li, J	2013	China	FFPE	ESCC	249	I–IV	NG	Archive	ANT	136/113	≤60, 105; >60, 144	qRT-PCR	SYBR	18s	NG	SC	OS
	Mathe	2009	Maryland	Frozen	AD	73	0–IV	45/27/1	During resection	ANT	63/10	<62, 39; ≥62, 34	qRT-PCR	TaqMan	U66	Median	R	OS
			Canada	Frozen	AD	27	0–IV	0/27/0	During resection	ANT	26/1	<62, 12; ≥62, 15	qRT-PCR	TaqMan	U66	Median	R	OS
			Maryland	Frozen	ESCC	24	0–IV	14/9/1	During resection	ANT	12/12	<62, 11; ≥62, 13	qRT-PCR	TaqMan	U66	Median	R	OS
			Japan	Frozen	ESCC	33	0–IV	15/18/0	During resection	ANT	30/3	<62, 14; ≥62, 19	qRT-PCR	TaqMan	U66	Median	R	OS
			Cornell	Frozen	ESCC	13	0–IV	4/9/0	During resection	ANT	10/3	<62, 3; ≥62, 10	qRT-PCR	TaqMan	U66	Median	R	OS
	Nguyen	2010	US/Canada	Frozen	AD	93	0–IV	40/53/0	During resection	ANT	83/10	61.7, 93	qRT-PCR	TaqMan	U66	Median	R	OS
	Wu	2014	China	Serum	ESCC	194	I–IV	0/194/0	Prior to surgery	normal	115/79	61.4, 194	qRT-PCR	TaqMan	U6	Mean	R	OS
miR-21	Hamano	2011	Japan	FFPE	ESCC	98	I–IV	98/0/0	Archive	ANT	84/14	High: 63.2 ± 8.5; Low: 60.0 ± 8.6	qRT-PCR	TaqMan	U48	Median	SC	OS
	Hu	2011	USA	FFPE	AD	158	0–IV	0/158/0	Archive	ANT	127/31	28–82, median 64	ISH	NG	NG	NG	R	OS/DFS
	Komatsu	2013	Japan	Plasma	ESCC	50	I–IV	0/50/0	Prior to surgery	normal	44/6	≥65, 25; <65, 25	qRT-PCR	TaqMan	U6	Normal	R	OS
	Li, P	2013	China	Frozen	ESCC	76	I–IV	NG	During resection	Normal	61/15	≥65, 29; <65, 47	qRT-PCR	SYBR	U6	5-fold	SC	DFS
	Mathe	2009	Maryland	Frozen	AD	73	0–IV	45/27/1	During resection	ANT	63/10	<62, 39; ≥62, 34	qRT-PCR	TaqMan	U66	Median	R	OS
			Canada	Frozen	AD	27	0–IV	0/27/0	During resection	ANT	26/1	<62, 12; ≥62, 15	qRT-PCR	TaqMan	U66	Median	R	OS
			Maryland	Frozen	ESCC	24	0–IV	14/9/1	During resection	ANT	12/12	<62, 11; ≥62, 13	qRT-PCR	TaqMan	U66	Median	R	OS
			Japan	Frozen	ESCC	33	0–IV	15/18/0	During resection	ANT	30/3	<62, 14; ≥62, 19	qRT-PCR	TaqMan	U66	Median	R	OS
			Cornell	Frozen	ESCC	13	0–IV	4/9/0	During resection	ANT	10/3	<62, 3; ≥62, 10	qRT-PCR	TaqMan	U66	Median	R	OS
	Tanaka	2013	Japan	Serum	ESCC	64	I–IV	0/64/0	Prior to surgery	Normal	49/15	≥65, 42; <65, 22	qRT-PCR	TaqMan	miR-39	Median	SC	PFS
	Zhao	2013	China	Frozen	ESCC	178	I–III	0/178/0	During resection	ANT	108/70	34–78, mean 62.2	qRT-PCR	TaqMan	U66	Median	SC	OS
miR-145	Feber	2011	US	Frozen	AD	45	I–IV	0/45/0	During resection	ANT	38/7	NG	qRT-PCR	NG	U6	Median	SC	OS
	Hamano	2011	Japan	FFPE	ESCC	98	I–IV	98/0/0	Archive	ANT	84/14	High: 63.2 ± 8.5; Low: 60.0 ± 8.6	qRT-PCR	TaqMan	U48	Median	SC	OS
	Ko	2012	Canada	FFPE	EC	25	III	0/25/0	Archive	PS	NG	NG	Microarray	BeadChip	NG	Median	SC	DFS
	Tanaka	2013	Japan	Serum	ESCC	64	I–IV	0/64/0	Prior to surgery	Normal	49/15	≥65, 42; <65, 22	qRT-PCR	TaqMan	miR-39	Median	SC	PFS

FFPE, formalin-fixed paraffin-embedded; PT (Y/N/U), preoperative treatment (yes, no, unknown); PS, posttreatment specimens; ISH, in situ hybridization; ANT, adjacent noncancerous tissue; R, reported; SC, survival curve; NG, not given; OS, overall survival; DFS, disease-free survival; PFS, progression-free survival.

## Abbreviations

EC: Esophageal cancer
AD: Esophageal adenocarcinoma
ESCC: Esophageal squamous cell carcinoma
miRNAs: MicroRNAs
OS: Overall survival
CI: Confidence interval
HR: Hazard ratios.
